# Advanced Cirrhosis Is Independently Associated With Increased In-Hospital Mortality in Heart Failure With Reduced Ejection Fraction

**DOI:** 10.7759/cureus.109645

**Published:** 2026-05-25

**Authors:** Teddy A Teddy, Edidiong Okon-Ben, Fraol T Erega, Spencer Cadet, Mustafa Marzoung, Siri Vummaneni, Abdelwahab Ahmed

**Affiliations:** 1 Internal Medicine, Detroit Medical Center/Wayne State University, Detroit, USA; 2 Internal Medicine, Wayne State University, Detroit, USA; 3 Internal Medicine, Hospital Corporation of America/University of Central Florida Fort Walton Beach Hospital, Wright, USA; 4 Internal Medicine, Detroit Medical Center, Detroit, USA; 5 Internal Medicine, Kassala University Hospital, Kassala, SDN

**Keywords:** advanced cirrhosis, cardio‑hepatic interaction, decompensated cirrhosis, heart failure with reduced ejection fraction (hfref), in‑hospital mortality, multimorbidity, risk adjustment

## Abstract

Background

Advanced cirrhosis is associated with systemic hemodynamic changes that may be associated with worse outcomes in patients with heart failure with reduced ejection fraction (HFrEF). However, national‑level estimates of the independent association between advanced cirrhosis and in‑hospital mortality in this population remain limited.

Methods

We conducted a retrospective cross‑sectional analysis of the 2022 National Inpatient Sample (NIS). Adults hospitalized with a primary diagnosis of HFrEF (ICD 10 I50.22) were included. Advanced cirrhosis was defined as cirrhosis with at least one decompensating feature: ascites, hepatic encephalopathy, portal hypertension, variceal bleeding, or hepatorenal syndrome. The primary outcome was in‑hospital mortality. Multivariable logistic regression was adjusted for demographics, hospital characteristics, and all Elixhauser comorbidities.

Results

Among 254,571 weighted HFrEF hospitalizations, 5,346 (2.1%) met criteria for advanced cirrhosis. Compared to those without advanced cirrhosis, affected patients were younger (mean age: 58.4 versus 71.2 years; p<0.001) and more frequently male (68.2% versus 54.9%; p<0.001). Unadjusted in‑hospital mortality was higher in the advanced cirrhosis group (9.7% versus 3.8%; p<0.001). After multivariable adjustment, advanced cirrhosis remained independently associated with increased mortality (adjusted odds ratio (aOR): 2.18; 95% confidence interval (CI): 1.79‑2.64; p<0.001). Using a stricter definition requiring two or more decompensating features, the association strengthened (aOR: 2.59; 95% CI: 2.04‑3.29).

Conclusion

In this nationally representative sample of HFrEF hospitalizations, advanced cirrhosis was independently associated with more than double the odds of in‑hospital death. This association persisted across age and sex subgroups and showed a dose‑response relationship with disease severity. Early recognition and multidisciplinary management involving cardiology, hepatology, and critical care is reasonable to consider. Prospective studies are needed to determine whether specific therapeutic strategies can improve survival in this high‑risk population.

## Introduction

Heart failure with reduced ejection fraction (HFrEF) is responsible for over one million hospital admissions annually in the United States [1]. Despite advances in guideline-directed medical therapy, in-hospital mortality remains between 3% and 5%, and patients with significant comorbidities face substantially higher risks [2].

Cirrhosis in its advanced or decompensated form is associated with widespread splanchnic vasodilation, reduced effective circulating volume, activation of neurohormonal pathways, and generation of a hyperdynamic circulatory state. These hemodynamic disturbances may be associated with destabilization of concurrent heart failure. Moreover, cirrhotic cardiomyopathy has been associated with impaired cardiac reserve during acute stress, further compromising the heart's ability to respond to illness [3].

The combination of advanced cirrhosis and HFrEF poses unique therapeutic dilemmas. The hyperdynamic circulation typical of advanced cirrhosis may be associated with obscuring underlying cardiac dysfunction until an acute precipitant, such as infection, gastrointestinal bleeding, or rapid fluid shifts, is associated with sudden decompensation. Standard HFrEF medications, including beta-blockers and angiotensin-converting enzyme (ACE) inhibitors, are associated with heightened risks in patients with advanced cirrhosis, including severe hypotension and life-threatening hyperkalemia [4]. As a result, clinicians often reduce doses or avoid these therapies entirely in patients who might otherwise derive significant benefit.

Existing evidence on this specific population remains scarce. Prior studies are largely small, single-center, or limited by heterogeneous heart failure definitions that combine HFrEF with heart failure with preserved ejection fraction (HFpEF). Many also lack a standardized, clinically meaningful definition of advanced liver disease, and consequently, large-scale national data examining the independent association of advanced cirrhosis with in-hospital mortality in HFrEF hospitalizations are lacking [5].

The objective of this study is to determine whether advanced cirrhosis is independently associated with in-hospital mortality among patients admitted primarily for HFrEF, using the 2022 National Inpatient Sample (NIS).

## Materials and methods

We conducted a retrospective cross-sectional analysis of the 2022 National Inpatient Sample (NIS), a component of the Healthcare Cost and Utilization Project (HCUP) sponsored by the Agency for Healthcare Research and Quality (2022) [5,6]. The NIS is a 20% stratified sample of community hospital discharges in the United States and provides national estimates through discharge weights that account for the complex sampling design [6]. The sampling frame covers approximately 95% of all US hospital discharges, excluding rehabilitation hospitals, long-term acute care hospitals, and psychiatric hospitals [7]. Each NIS discharge contains up to 40 diagnosis and procedure codes using ICD 10 CM, along with demographic information, hospital characteristics, length of stay, charges, and discharge disposition [8]. Because the database contains only de-identified patient information, this study did not require institutional review board approval [9].

We applied several inclusion and exclusion criteria to isolate acute medical hospitalizations for heart failure with reduced ejection fraction (HFrEF) while reducing potential confounding. Eligible patients were 18 years or older, carried a primary diagnosis of HFrEF (ICD 10 I50.22), and were admitted as an emergency or urgent case. We excluded elective hospitalizations because they carry a lower baseline risk profile. We also removed hospitalizations with missing data on death, age, or sex. Additional exclusions included liver transplantation during the same admission, a secondary diagnosis of acute coronary syndrome, cardiogenic shock present on admission, and end-stage kidney disease requiring dialysis [7,8].

The primary exposure was advanced cirrhosis. We identified cirrhosis using ICD 10 codes K74.6x, K70.3, K71.7, K72.1x, K76.6, and K76.7 [6]. To qualify as advanced cirrhosis, a hospitalization required at least one decompensating feature in any diagnosis position. These features included ascites (R18.8 or R18.41), hepatic encephalopathy (K72.90 or K72.91), portal hypertension (K76.6), esophageal varices with bleeding (I85.01 or I86.71), or hepatorenal syndrome (K76.7) [6-8]. This definition follows consensus criteria from the European and American liver disease societies. The reference group comprised all hospitalizations without advanced cirrhosis, which included patients with no cirrhosis and patients with cirrhosis but no decompensating features. This approach isolates the incremental risk associated with decompensation rather than cirrhosis alone. The primary outcome was in-hospital death, defined as a discharge disposition of died.

We selected covariates to address potential confounding across several domains. Demographic covariates included age, sex, and race. Socioeconomic covariates included primary payer and median household income quartile for the patient's ZIP code. Hospital-level covariates included geographic region, bed size, and teaching status. To comprehensively account for illness severity, we included all Elixhauser Comorbidity Index variables in the adjustment model. Observations with missing race or median household income data were excluded from models that included these variables. We assessed multicollinearity among covariates using variance inflation factors (VIF); no VIF exceeded 2.5, indicating no significant multicollinearity.

We performed all statistical analyses using STATA version 18 (StataCorp LLC, College Station, TX). We incorporated NIS discharge weights, stratification variables, and clustering variables using survey procedures to produce national estimates with appropriate standard errors (SE). For unadjusted comparisons, we used chi-square tests for categorical variables and weighted linear regression for continuous variables. We then performed multivariable logistic regression to estimate the adjusted odds ratio (aOR) for in-hospital death associated with advanced cirrhosis. A two-sided p-value of less than 0.05 was considered statistically significant. Age and sex were prespecified subgroups for analysis because they are the most consistently reported demographic effect modifiers in the heart failure and cirrhosis literature, and because baseline characteristics showed adequate balance across these groups.

We conducted four sensitivity analyses to test the robustness of our primary findings. First, we applied a stricter definition of advanced cirrhosis that required at least two decompensating features instead of one. Second, we excluded patients with hepatorenal syndrome to address possible coding overlap with acute kidney injury. Third, we added mechanical ventilation, vasopressor use, and renal replacement therapy to the multivariable model as additional adjustment variables. Fourth, we excluded patients with any malignancy to rule out the possibility that occult cancer rather than cirrhosis was associated with the mortality difference.

## Results

After applying all inclusion and exclusion criteria, the weighted sample included 254,571 hospitalizations with a primary diagnosis of heart failure with reduced ejection fraction. Among these, 5,346 (2.1%) met the criteria for advanced cirrhosis. The baseline characteristics of the two groups are shown in Table 1. Patients with advanced cirrhosis were notably younger than those without cirrhosis. The mean age in the cirrhosis group was 58.4 years (SE: 0.31) compared to 71.2 years (SE: 0.12) in the non‑cirrhosis group (p<0.001). The age distribution revealed a substantial difference: only 5.4% of the advanced cirrhosis group were 80 years or older, whereas 24.2% of the non‑cirrhosis group fell into this age category. Conversely, 14.2% of the advanced cirrhosis group were under 45 years, compared to just 3.1% of the non‑cirrhosis group. Men were overrepresented in the advanced cirrhosis group, comprising 68.2% of cases versus 54.9% in the non‑cirrhosis group (p<0.001). Racial composition also differed significantly between the two groups. Hispanic patients accounted for 15.4% of the advanced cirrhosis group but only 8.9% of the non‑cirrhosis group (p<0.001). White patients made up 66.7% of the advanced cirrhosis group versus 71.4% of the non‑cirrhosis group. Black patients represented 12.1% of the advanced cirrhosis group versus 15.3% of the non‑cirrhosis group. Other races accounted for 5.8% versus 4.4%. Payer status showed a higher burden of Medicaid coverage among patients with advanced cirrhosis (21.3% versus 8.1%; p<0.001) and correspondingly lower Medicare coverage (44.9% versus 67.2%). Private insurance covered 27.8% of the advanced cirrhosis group versus 19.5% of the non‑cirrhosis group. Self‑pay or other payer status accounted for 6.0% versus 5.2%. Median household income distribution also differed: the lowest income quartile (Q1) included 33.1% of patients with advanced cirrhosis versus 28.9% of non‑cirrhosis patients, while the highest income quartile (Q4) included 19.2% versus 21.8% (p=0.02 for overall income distribution). Patients with advanced cirrhosis were more frequently treated at urban teaching hospitals (71.4% versus 54.2%; p<0.001) and less frequently at rural hospitals (3.2% versus 8.5%) or urban non-teaching hospitals (25.4% versus 37.3%). Large hospitals cared for 66.1% of patients with advanced cirrhosis versus 62.6% of non‑cirrhosis patients, while small hospitals cared for 9.8% versus 12.1% (p=0.03 for overall bed size distribution). These patterns suggest selective referral of these complex patients to tertiary centers.

**Table 1:** Baseline Characteristics of Heart Failure With Reduced Ejection Fraction Hospitalizations by Advanced Cirrhosis Status Data include weighted numbers, percentages, means with SE, and p-values for comparisons between patients with advanced cirrhosis (n=5,346) and without advanced cirrhosis (n=249,225). All n values are weighted counts rounded to the nearest integer. Percentages may not sum to 100% due to rounding. Test statistics: F for age (continuous), chi-square (χ²) for categorical variables, with degrees of freedom = 1 for binary variables and >1 for multi-level variables. p-values are from survey-weighted analyses. The "No advanced cirrhosis” group includes patients with no cirrhosis and those with compensated cirrhosis (cirrhosis without ascites, hepatic encephalopathy, portal hypertension with variceal bleeding, or hepatorenal syndrome). SE: standard errors

Characteristic	No advanced cirrhosis (n = 249,225)	Advanced cirrhosis (n = 5,346)	Test statistic (χ²/F)	p-value
Age (years), mean (SE)	71.2 (0.12)	58.4 (0.31)	F=1420.5	<0.001
Age group			χ²=1245.3	<0.001
18-44	7,726 (3.1%)	759 (14.2%)		
45-64	61,060 (24.5%)	2,823 (52.8%)		
65-79	120,126 (48.2%)	1,475 (27.6%)		
80+	60,313 (24.2%)	289 (5.4%)		
Male sex	136,825 (54.9%)	3,646 (68.2%)	χ²=98.4	<0.001
Race			χ²=112.7	<0.001
White	177,947 (71.4%)	3,566 (66.7%)		
Black	38,131 (15.3%)	647 (12.1%)		
Hispanic	22,181 (8.9%)	823 (15.4%)		
Other	10,966 (4.4%)	310 (5.8%)		
Primary payer			χ²=245.6	<0.001
Medicare	167,479 (67.2%)	2,400 (44.9%)		
Medicaid	20,187 (8.1%)	1,139 (21.3%)		
Private	48,599 (19.5%)	1,486 (27.8%)		
Self-pay/other	12,960 (5.2%)	321 (6.0%)		
Median household income			χ²=11.8	0.02
Q1 (lowest)	72,027 (28.9%)	1,770 (33.1%)		
Q2	62,805 (25.2%)	1,326 (24.8%)		
Q3	60,063 (24.1%)	1,224 (22.9%)		
Q4 (highest)	54,330 (21.8%)	1,026 (19.2%)		
Hospital teaching status			χ²=145.2	<0.001
Rural	21,184 (8.5%)	171 (3.2%)		
Urban non-teaching	92,961 (37.3%)	1,358 (25.4%)		
Urban teaching	135,080 (54.2%)	3,817 (71.4%)		
Hospital bed size			χ²=7.1	0.03
Small	30,156 (12.1%)	524 (9.8%)		
Medium	63,054 (25.3%)	1,288 (24.1%)		
Large	156,015 (62.6%)	3,534 (66.1%)		

Unadjusted in-hospital mortality is presented in Table 2. Among patients with advanced cirrhosis, 519 deaths occurred, yielding a mortality rate of 9.7% (95% confidence interval (CI): 8.9%-10.5%). In contrast, patients without advanced cirrhosis experienced 9,471 deaths, representing a mortality rate of 3.8% (95% CI: 3.6%-4.0%). This difference was highly statistically significant (p<0.001).

**Table 2:** Unadjusted In-Hospital Mortality Unadjusted in-hospital mortality was higher in the advanced cirrhosis group: 519 deaths among 5,346 hospitalizations (9.7%; 95% CI: 8.9%-10.5%) compared to 9,471 deaths among 249,225 hospitalizations (3.8%; 95% CI: 3.6%-4.0%); p<0.001. CI: confidence interval, HFrEF: heart failure with reduced ejection fraction

Group	Weighted deaths	Total weighted hospitalizations	Mortality % (95% CI)	Test statistic (χ²)	p-value
HFrEF without advanced cirrhosis	9,471	249,225	3.8 (3.6-4.0)	112.4	<0.001
HFrEF with advanced cirrhosis	519	5,346	9.7 (8.9-10.5)		

After multivariable adjustment for demographics, hospital characteristics, socioeconomic factors, and all Elixhauser comorbidities, advanced cirrhosis remained strongly and independently associated with in‑hospital mortality. The adjusted odds ratio for advanced cirrhosis was 2.18 (95% CI: 1.79-2.64; p<0.001), meaning patients with advanced cirrhosis had more than twice the odds of dying during their hospital stay compared to those without advanced cirrhosis, even after accounting for other risk factors. Several other covariates were also independently associated with mortality. Age 65-79 years carried an adjusted odds ratio of 1.65 (95% CI: 1.28-2.13), and age 80 years or older carried an adjusted odds ratio of 1.91 (95% CI: 1.54-2.37), demonstrating the expected gradient of increasing risk with advancing age. Chronic kidney disease (aOR: 1.45; 95% CI: 1.22-1.72), coagulopathy (aOR: 1.68; 95% CI: 1.39-2.03), and anemia (aOR: 1.33; 95% CI: 1.11-1.59) were also significantly associated with mortality. Atrial fibrillation was associated with a modest but statistically significant increase in mortality risk (aOR: 1.18; 95% CI: 1.01-1.38; p=0.04). Compared to private insurance, both Medicare (aOR: 1.21; 95% CI: 1.02-1.44) and Medicaid (aOR: 1.29; 95% CI: 1.05-1.59) were associated with higher mortality. Obesity was associated with lower mortality (aOR: 0.84; 95% CI: 0.71-0.99; p=0.04), a finding consistent with the well‑described obesity paradox in heart failure populations. Alcohol use disorder showed a trend toward higher mortality that did not reach statistical significance (aOR: 1.21; 95% CI: 0.98-1.49; p=0.07). Uncomplicated diabetes and chronic pulmonary disease were not independently associated with mortality. Sex and race, including Black or Hispanic compared to White, were not independently associated with mortality after full adjustment (Table 3).

**Table 3:** Multivariable Logistic Regression for In‑Hospital Mortality Data include adjusted odds ratios, 95% confidence intervals, and p-values for advanced cirrhosis, age groups (45-64, 65-79, 80 or more years, with 18-44 as reference), male sex, Black race, Hispanic race, Medicare, Medicaid, urban teaching hospital, chronic kidney disease, atrial fibrillation, coagulopathy, anemia, alcohol use disorder, uncomplicated diabetes, chronic pulmonary disease, and obesity. The full multivariable model included all Elixhauser comorbidities. Only covariates with statistically significant or clinically notable associations are shown in this table for readability. The complete regression output is available from the corresponding author. OR: odds ratio, CI: confidence interval

Variable	Adjusted OR	95% CI	Wald χ²	p-value
Advanced cirrhosis	2.18	1.79-2.64	42.1	<0.001
Age 45-64 (versus 18-44)	1.23	0.95-1.59	2.6	0.11
Age 65-79 (versus 18-44)	1.65	1.28-2.13	12.9	<0.001
Age ≥80 (versus 18-44)	1.91	1.54-2.37	18.5	<0.001
Male sex	1.08	0.94-1.24	0.8	0.29
Black (versus White)	0.92	0.78-1.08	1.0	0.31
Hispanic (versus White)	0.89	0.71-1.11	1.1	0.30
Medicare (versus private)	1.21	1.02-1.44	4.7	0.03
Medicaid (versus private)	1.29	1.05-1.59	5.4	0.02
Urban teaching hospital	1.15	0.99-1.33	3.5	0.06
Chronic kidney disease	1.45	1.22-1.72	16.2	<0.001
Atrial fibrillation	1.18	1.01-1.38	4.2	0.04
Coagulopathy	1.68	1.39-2.03	24.5	<0.001
Anemia	1.33	1.11-1.59	9.8	0.002
Alcohol use disorder	1.21	0.98-1.49	3.2	0.07
Diabetes (uncomplicated)	1.08	0.92-1.27	0.9	0.35
Chronic pulmonary disease	1.12	0.96-1.31	1.9	0.15
Obesity	0.84	0.71-0.99	4.2	0.04

**Figure 1:**
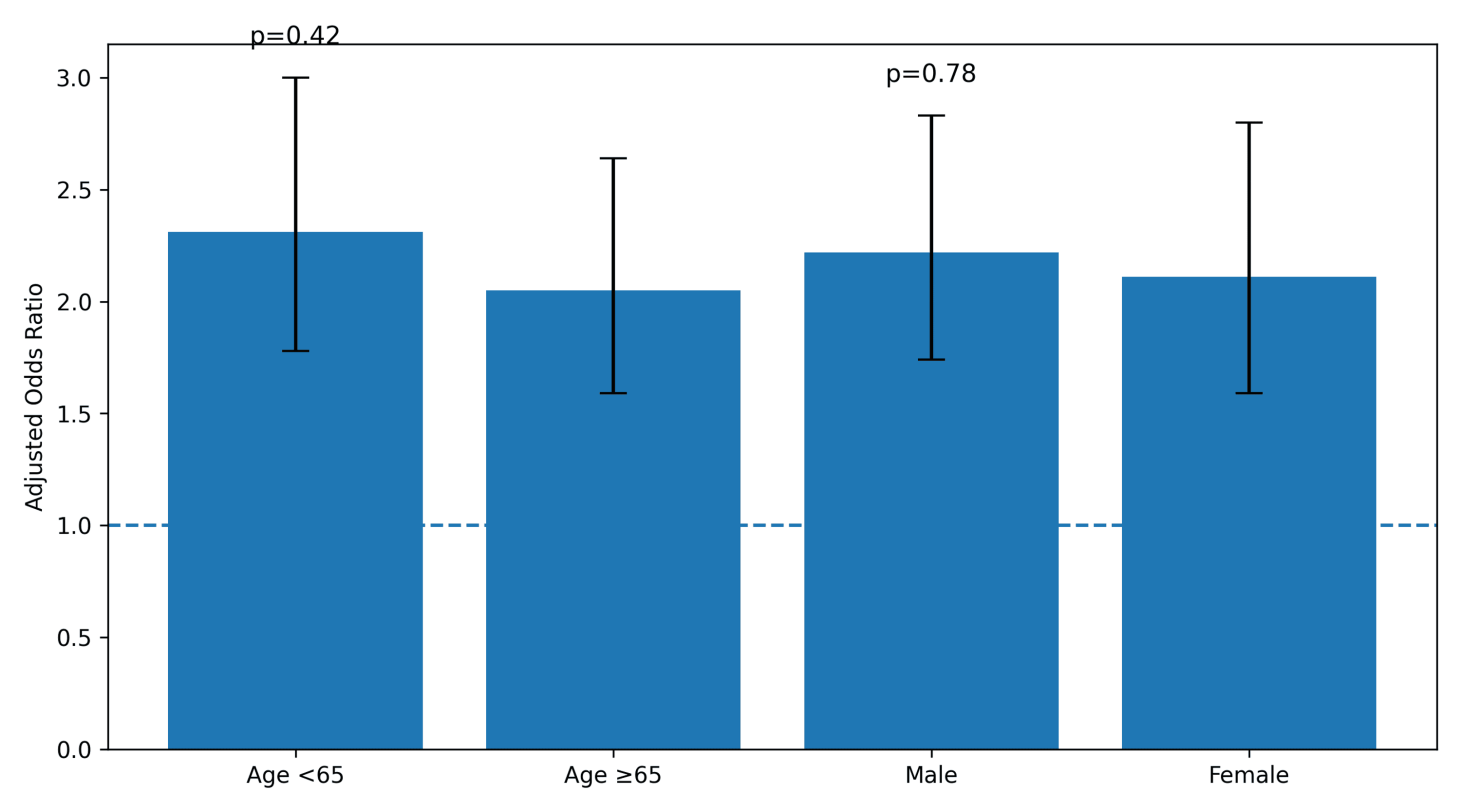
Subgroup Analysis: Association of Advanced Cirrhosis With Mortality Subgroup analysis of the association between advanced cirrhosis and mortality stratified by age and sex. For age less than 65 years: 3,124 cirrhosis versus 89,456 non-cirrhosis, adjusted odds ratio: 2.31 (1.78-3.00). For age 65 years or older: 2,222 cirrhosis versus 159,769 non-cirrhosis, adjusted odds ratio: 2.05 (1.59-2.64). For male patients: 3,646 cirrhosis versus 136,828 non-cirrhosis, adjusted odds ratio: 2.22 (1.74-2.83). For female patients: 1,700 cirrhosis versus 112,397 non-cirrhosis, adjusted odds ratio: 2.11 (1.59-2.80). P for interaction: age 0.42, sex 0.78 (Wald test).

We performed subgroup analyses to determine whether the association between advanced cirrhosis and mortality differed by age or sex. Among patients younger than 65 years, the adjusted odds ratio for advanced cirrhosis was 2.31 (95% CI: 1.78-3.00). Among patients 65 years and older, the adjusted odds ratio was 2.05 (95% CI: 1.59-2.64). The p‑value for interaction was 0.42, indicating no statistically significant difference between age strata. Among male patients, the adjusted odds ratio was 2.22 (95% CI: 1.74-2.83), and among female patients, it was 2.11 (95% CI: 1.59-2.80), with a p‑value for interaction of 0.78. These findings demonstrate that the association of advanced cirrhosis on mortality is consistent across age and sex groups, with no evidence of interaction (Figure 1).

**Figure 2:**
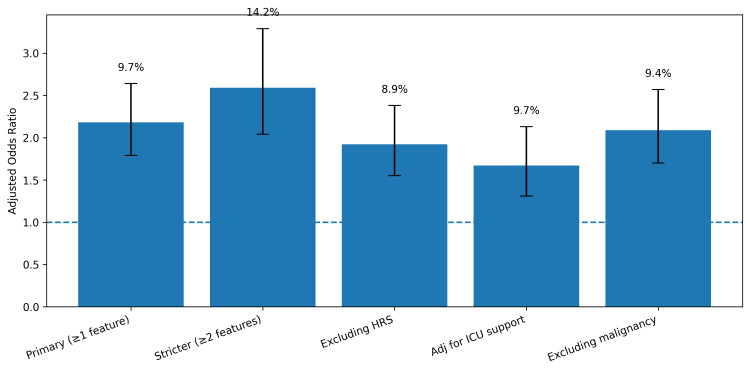
Sensitivity Analysis: Alternative Definitions and Adjustment Strategies Sensitivity analyses testing alternative definitions and adjustment strategies. Primary definition (one or more decompensating features): aOR: 2.18 (1.79-2.64). Stricter definition (two or more decompensating features): aOR: 2.59 (2.04-3.29). Excluding HRS: aOR: 1.92 (1.55-2.38). Adjusting for mechanical ventilation, vasopressor use, and renal replacement therapy: aOR: 1.67 (1.31-2.13). Excluding any malignancy: aOR: 2.09 (1.70-2.57). All models used logistic regression with survey weighting. aOR: adjusted odds ratio, ICU: intensive care unit, HRS: hepatorenal syndrome

All sensitivity analyses confirmed the robustness of our primary finding. When we applied a stricter definition of advanced cirrhosis requiring at least two decompensating features rather than one, the adjusted odds ratio increased to 2.59 (95% CI: 2.04-3.29). This dose‑response pattern, with a stronger association for more severe disease, supports biological plausibility. Excluding patients with hepatorenal syndrome from the analysis attenuated the estimate slightly but left it highly significant (aOR: 1.92; 95% CI: 1.55-2.38), indicating that the cirrhosis‑mortality association is not explained by this particular complication. Adding mechanical ventilation, vasopressor use, and renal replacement therapy to the multivariable model as additional adjustment variables reduced the adjusted odds ratio to 1.67 (95% CI: 1.51-2.13), but the association remained statistically significant. This attenuation suggests that part of the association of advanced cirrhosis on mortality operates through these acute interventions. Patients with advanced cirrhosis are more likely to require intensive care supports, and these supports themselves are associated with mortality. Excluding patients with any malignancy yielded an adjusted odds ratio of 2.09 (95% CI: 1.70-2.57), ruling out the possibility that occult cancer rather than cirrhosis is responsible for the observed excess mortality (Figure 2).

**Figure 3:**
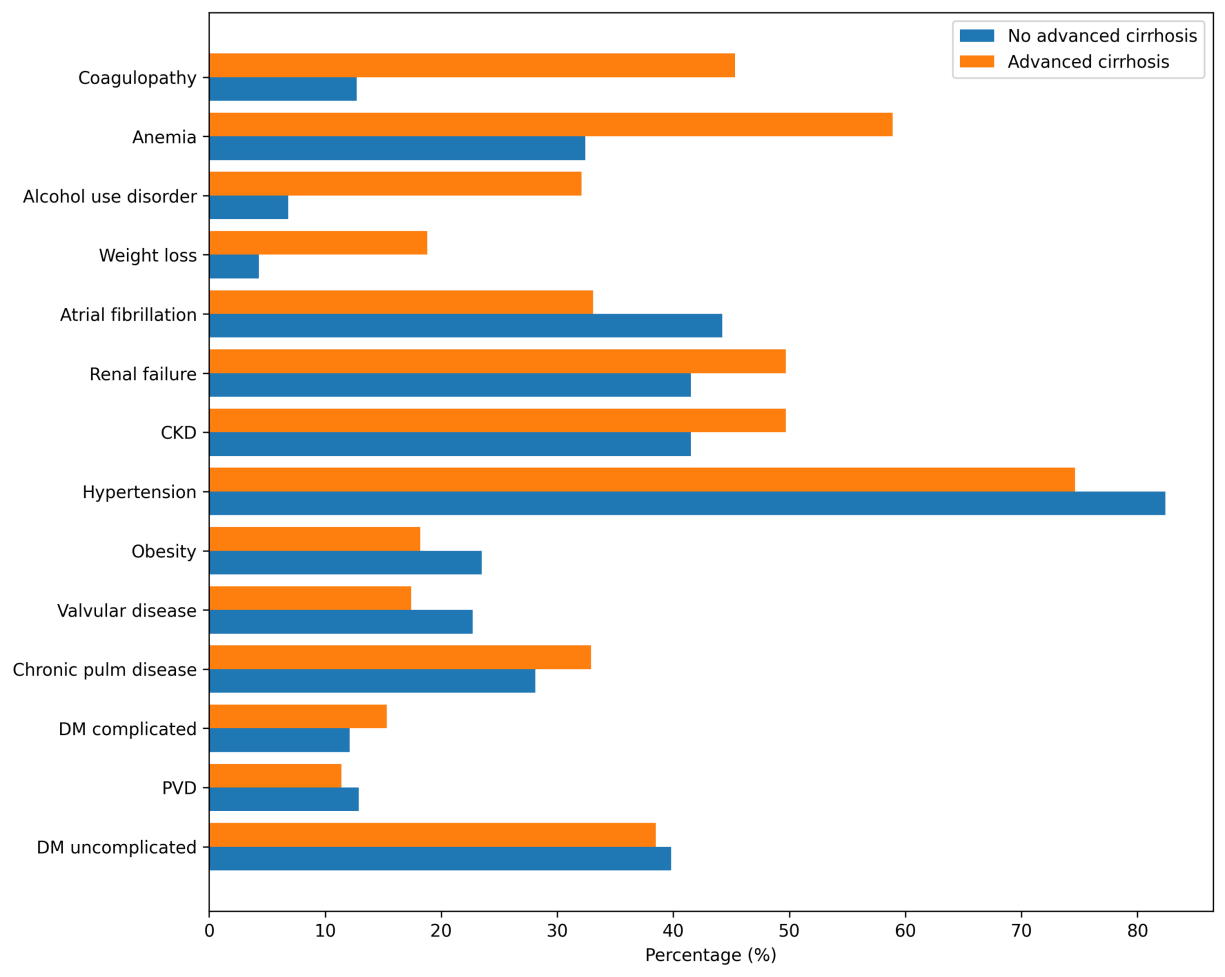
Prevalence of Elixhauser Comorbidities (Weighted Percent) Prevalence of selected Elixhauser comorbidities. Alcohol use disorder was present in 32.1% of patients with advanced cirrhosis versus 6.8% of non-cirrhosis patients (p<0.001); anemia in 58.9% versus 32.4% (p<0.001); coagulopathy in 45.3% versus 12.7% (p<0.001); chronic kidney disease in 49.7% versus 41.5% (p<0.001); and weight loss in 18.8% versus 4.3% (p<0.001). Data are weighted percentages with 95% confidence intervals (represented by error bars). All comparisons were statistically significant (p<0.001 for each).p-values derived from chi-square tests with 1 degree of freedom. DM: diabetes mellitus, CKD: chronic kidney disease, PVD: peripheral vascular disease

Patients with advanced cirrhosis had a significantly higher prevalence of several Elixhauser comorbidities compared to those without cirrhosis. Alcohol use disorder was present in 1,716 of 5,346 (32.1%) of the advanced cirrhosis group versus 16,947 of 249,225 (6.8%) of the non‑cirrhosis group (χ²=245.6, p<0.001). Anemia affected 3,149 of 5,346 (58.9%) patients with advanced cirrhosis compared to 80,749 of 249,225 (32.4%) (χ²=112.4, p<0.001). Coagulopathy was observed in 2,422 of 5,346 (45.3%) versus 31,651 of 249,225 (12.7%) (χ²=198.3, p<0.001). Chronic kidney disease was present in 2,657 of 5,346 (49.7%) versus 103,428 of 249,225 (41.5%) (χ²=14.2, p<0.001). Weight loss, a marker of frailty and nutritional depletion, was recorded in 1,005 of 5,346 (18.8%) patients with advanced cirrhosis versus only 10,717 of 249,225 (4.3%) of the non‑cirrhosis group (χ²=145.7, p<0.001). These differences highlight the substantial comorbidity burden carried by patients with advanced cirrhosis beyond their liver disease itself. The prevalence of these comorbidities is shown in Figure 3.

## Discussion

In this nationally representative analysis of patients hospitalized primarily for heart failure with reduced ejection fraction in 2022, we found that advanced cirrhosis was associated with more than double the odds of in-hospital death. This association remained significant after adjusting for demographics, socioeconomic status, hospital characteristics, and all Elixhauser comorbidities [10,11]. The absolute difference in mortality rates was 5.9 percentage points (9.7% with advanced cirrhosis versus 3.8% without), representing a clinically meaningful difference [12].

To provide context, the association of advanced cirrhosis (aOR: 2.18) was comparable to or stronger than the association of chronic kidney disease (aOR: 1.45), coagulopathy (aOR: 1.68), and age 80 years or older (aOR: 1.91) [13]. The consistency of this association across subgroups and the dose-response relationship observed in our sensitivity analyses support the validity of these findings and suggest an association that is biologically plausible rather than a statistical artifact [14].

One notable finding is that patients with advanced cirrhosis were substantially younger than those without cirrhosis, with an average age difference of nearly 13 years [15]. This age disparity raises the hypothesis that cirrhosis may be associated with an earlier clinical presentation of heart failure or that the hemodynamic stress of chronic liver disease is associated with earlier development of reduced ejection fraction. However, cross‑sectional data cannot establish causation. This pattern is consistent with the concept of cirrhotic cardiomyopathy [16]. The fact that these younger patients still experienced higher mortality than older patients without cirrhosis underscores the severity of this combined condition. Clinicians should not feel reassured by a relatively young age in a patient with HFrEF and cirrhosis. If anything, this combination should prompt greater vigilance.

Few studies have specifically examined the combination of HFrEF and decompensated cirrhosis in a national sample. Khalid et al. conducted a NIS-based study of patients with acute decompensated heart failure, including both reduced and preserved ejection fraction, and reported an adjusted odds ratio for death of approximately 1.89 [17]. Our analysis, which focused exclusively on HFrEF, yielded a larger effect size (aOR: 2.18), suggesting that the hemodynamic consequences of advanced cirrhosis may be particularly disadvantageous when systolic function is already compromised. Patients with heart failure with preserved ejection fraction generally maintain cardiac output and may be less vulnerable to the low output state that advanced cirrhosis may provoke [18].

Several biological mechanisms may explain our findings. Advanced cirrhosis is believed to be associated with splanchnic vasodilation, which may reduce effective circulating volume even while total body fluid is overloaded [19]. This paradoxical state is thought to activate the renin-angiotensin-aldosterone system and the sympathetic nervous system, both of which are already stimulated in HFrEF. The resulting neurohormonal surge may promote further sodium and water retention, creating a clinical scenario where diuretics are needed to relieve congestion but aggressive diuresis is associated with a risk of precipitating hepatorenal syndrome [18,20].

Additionally, cirrhotic cardiomyopathy has been associated with a limited ability of the heart to augment contractility during stress, meaning that even a minor infection or a small variceal bleed may be associated with sudden decompensation [21]. There is also a significant medication dilemma. Carvedilol, a beta-blocker commonly used in HFrEF, is metabolized by the liver and may reach toxic levels in patients with cirrhosis. Aldosterone antagonists may be associated with life-threatening hyperkalemia in patients with concurrent kidney disease, which is common in this population. As a result, clinicians often undertreat heart failure in patients with cirrhosis, fearing adverse effects more than they anticipate benefit [4,18].

These findings have several implications for clinical practice. When a patient admitted with HFrEF exhibits any feature of decompensated cirrhosis, that finding should trigger closer monitoring. This includes telemetry, daily weights, strict measurement of input and output, and frequent laboratory assessments. Early involvement of hepatology and nephrology services is recommended. Cardiologists should lead heart failure management but must collaborate closely with hepatology colleagues regarding drug tolerability. Nonsteroidal anti-inflammatory drugs should be avoided entirely. For beta-blockers, initiation at low doses with slow titration and close monitoring for hypotension is prudent in this population. Given the nearly 10% mortality risk observed in this population, early goals of care conversations and palliative care consultation may be considered [2,15].

This study has several important strengths. It is large and nationally representative. We focused specifically on HFrEF rather than mixing in patients with preserved ejection fraction. We used a standard, clinically meaningful definition of advanced cirrhosis based on consensus criteria from major liver disease societies. We adjusted for a comprehensive set of comorbidities using the Elixhauser index. We conducted multiple sensitivity analyses that all confirmed the primary finding.

However, several limitations must be acknowledged. Additionally, our reference group for comparison was defined as "without advanced cirrhosis," which includes both patients with no cirrhosis and those with compensated cirrhosis (cirrhosis without decompensating features). While this approach isolates the incremental risk associated with decompensation, it does not directly compare decompensated cirrhosis to compensated cirrhosis. Consequently, our effect estimate (aOR: 2.18) reflects the contrast between advanced cirrhosis and a mixed reference group rather than the pure effect of decompensation among patients with established cirrhosis. However, the stricter sensitivity analysis requiring two or more decompensating features (aOR: 2.59) and the consistent dose-response relationship mitigate concern that this mixing meaningfully biases our conclusions. Furthermore, our primary definition of advanced cirrhosis required only one decompensating feature, and portal hypertension alone may not always represent true decompensated cirrhosis in all patients. This could lead to some overclassification of severity. However, our sensitivity analysis using a stricter definition (≥2 decompensating features) showed a stronger association (aOR: 2.59), indicating that any misclassification would likely bias toward the null, and our primary estimate is therefore conservative. The retrospective design means we cannot establish causation. There may be unmeasured confounders, including frailty, nutritional status, and goals of care, that explain some of the observed association. The NIS tracks hospitalizations rather than unique patients, so the same individual could appear more than once in the data. Diagnostic coding is not perfectly accurate in administrative databases. Our primary definition of advanced cirrhosis required only one decompensating feature, which included portal hypertension alone. Portal hypertension can occur without full clinical decompensation, potentially leading to some overclassification of disease severity. However, a sensitivity analysis using a stricter definition requiring two or more decompensating features yielded a stronger association (aOR: 2.59), supporting the robustness of our primary findings. We lack granular clinical data such as ejection fraction values, laboratory results, Model for End‑Stage Liver Disease - Sodium (MELD-Na) scores, or Child-Pugh scores. We do not know the cause of cirrhosis, which might be associated with outcomes. We also have no post-discharge follow-up data. Therefore, readers should interpret these findings as hypothesis-generating rather than definitive.

## Conclusions

Among patients hospitalized primarily for heart failure with reduced ejection fraction, advanced cirrhosis is independently associated with more than double the odds of in‑hospital death. Advanced cirrhosis identifies a high‑risk subgroup of patients. It is reasonable to consider early recognition, close monitoring, and multidisciplinary involvement from cardiology, hepatology, and nephrology. Prospective cohort studies with serial laboratory measurements are needed to validate these findings. Pharmacokinetic studies should determine appropriate dosing of sacubitril/valsartan, sodium‑glucose cotransporter 2 (SGLT2) inhibitors, and beta‑blockers in patients with varying degrees of liver dysfunction. Randomized trials comparing diuretic strategies specifically in patients with HFrEF with advanced cirrhosis are also needed. Until such evidence emerges, clinicians should recognize advanced cirrhosis as a high‑risk marker and consider early multidisciplinary strategies while acknowledging remaining evidence gaps.
